# Stries angioïdes compliquées d'une néovascularisation choroïdienne

**DOI:** 10.11604/pamj.2014.19.312.5099

**Published:** 2014-11-21

**Authors:** Mohammed El Mellaoui, Abdelkader Laktaoui

**Affiliations:** 1 Service d'Ophtalmologie, Hôpital Militaire Moulay Ismaïl, Meknès, Maroc

**Keywords:** Stries angioïdes, éovascularisation choroïdienne, membrane de Bruch, Angioid streaks, choroidal neovascularization, Bruch membrane

## Image en medicine

Les stries angioïdes sont des lésions rares du fond d'oeil. Elles sont souvent associées à des pathologies générales telles le pseudoxanthome élastique, la drépanocytose, la maladie de Paget ou le syndrome de Ehlers Danlos. Elles correspondent à des ruptures de la membrane de Bruch, visibles sous forme de lignes radiaires, sombres ou rougeâtres partant de la papille et dont le risque évolutif potentiel est l’émergence de néovaisseaux choroïdiens. Notre patient est un homme de 43 ans sans antécédents pathologiques notables qui présente une acuité visuelle chiffrée à 3/10 P8 au niveau de l'oeil droit et à 1/20 P14 au niveau de l'oeil gauche. L'examen du segment antérieur est sans anomalies avec un tonus oculaire à 14 mmHg aux deux yeux. L'examen du fond d'oeil révèle la présence de stries angioïdes bilatérales compliquées d'une néovascularisation choroïdienne maculaire avec développement d'une fibrose sous rétinienne au niveau de l'oeil gauche. L'angiographie et la tomographie par cohérence optique confirment le diagnostic. La complication néovasculaire responsable de malvoyance est prise en charge essentiellement par les injections intravitréennes d'anti-vascular endothélial growth factor (VEGF) avec une efficacité prouvée. Le médecin ophtalmologiste doit connaître cette pathologie, afin d'orienter le patient vers une prise en charge systémique si nécessaire et afin d'instaurer un traitement ciblé et rapide en cas de complication néovasculaire.

**Figure 1:**
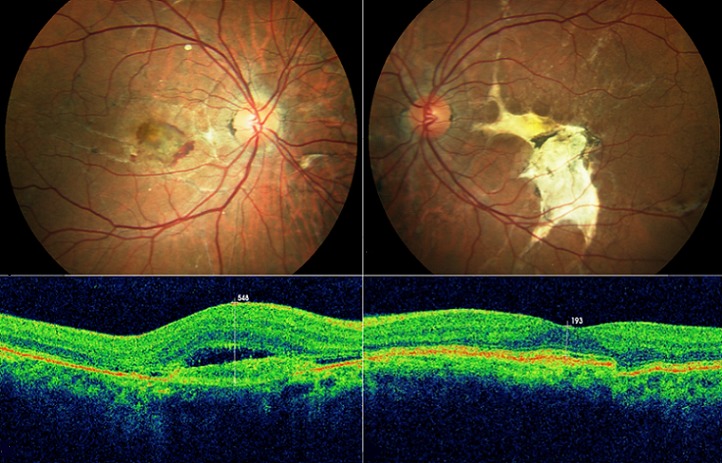
Stries angioïdes compliquées d'une néovascularisation choroïdienne au niveau de l'oeil droit et d'une fibrose sous rétinienne au niveau de l'oeil gauche

